# Serotype-Switch Variant of Multidrug-Resistant *Streptococcus pneumoniae* Sequence Type 271 

**DOI:** 10.3201/eid2706.203629

**Published:** 2021-06

**Authors:** Erin M. Scherer, Bernard Beall, Benjamin Metcalf

**Affiliations:** Centers for Disease Control and Prevention, Atlanta, Georgia, USA

**Keywords:** recombination, capsular switch, antimicrobial resistance, serotype 3, ST271 lineage, pneumococcal, respiratory infections, bacteria, vaccines, Streptococcus pneumoniae, streptococci, pneumococcus

## Abstract

We discovered 3 invasive, multidrug-resistant *Streptococcus pneumoniae* isolates of vaccine-refractory capsular serotype 3 that recently arose within the successful sequence type 271 complex through a serotype switch recombination event. Mapping genomic recombination sites within the serotype 3/sequence type 271 progeny revealed a 55.9-kb donated fragment that encompassed *cps3, pbp1a,* and additional virulence factors.

*Streptococcus pneumoniae* (pneumococcus) clonal complex (CC) 271 consists of broadly distributed, antimicrobial drug–resistant pneumococcal strains of serotypes 19F and 19A, first recorded in serotype 19F strains from 1992 (https://pubmlst.org). Two successful multivalent pneumococcal conjugate vaccines (PCV) targeting common invasive pneumococcal disease (IPD) serotypes (*1*,*2*) were introduced in the United States in 2000 (PCV7, targeting serotypes 4, 6B, 9V, 14, 18C, 19F, and 23F) and 2010 (PCV13, targeting PCV7 serotypes plus 1, 3, 5, 6A, 7F, and 19A). Serotype 19A CC271, likely arising through serotype switch with serotype 19F, emerged as the most common cause of IPD in the United States after introduction of PCV7 (*3*). After introduction of PCV13, IPD caused by serogroup 19 CC271 greatly decreased (*2*,*4*).

Three serotype 3 sequence type (ST) 271 pneumococcal isolates from adult invasive pneumonia cases in Connecticut and Maryland, USA, were recovered through the Centers for Disease Control and Prevention Active Bacterial Core surveillance (ABCs) in 2016 (isolate 20155315-S-ABC), 2017 (isolate 20170822-S-ABC), and 2018 (isolate 20182806-S-ABC). These isolates have a common origin; they differ by 6–29 single nucleotide polymorphisms (SNPs) and share identical serotype 3 capsular polysaccharide biosynthetic operons (*cps3*) and penicillin-binding protein (PBP) sequence types (1a-17/2b-16/2x-47). Isolates 20155315-S-ABC and 20170822-S-ABC differed by 6 SNPs and were recovered 16 months apart during 2016–2017 from the same person. These 3 pneumococcal isolates represent a novel recombinant serotype 3 variant of the globally distributed, antimicrobial drug–resistant lineage ST271.

The polysaccharide capsule, of which there are >90 structurally and serologically unique types, is the primary pneumococcal virulence factor (*5*). Serotype 3 is historically associated with higher virulence than other serotypes and currently causes the largest proportion of IPD cases in the United States (>12% of all cases) (*4*). Although serotype 3 is included in PCV13, PCV13 provides poor protection against serotype 3 because of unique qualities of the serotype 3 capsule (*6*,*7*). In keeping with the ST271 heritage, the 3 serotype 3/ST271 isolates share the same antimicrobial-resistance mechanisms, including reduced affinities for β-lactams (mosaic PBPs), dual mechanisms for macrolide resistance (ErmB rRNA methylase and MefA*/*MsrD macrolide efflux system), clindamycin resistance (ErmB), cotrimoxazole resistance (altered FolA and FolP enzymes), and tetracycline resistance (TetM*-*mediated ribosome alteration) (Appendix 1).

To reveal genomic regions within the 3 serotype 3/ST271 progeny resulting from recombination, we first identified likely recipient and donor strains involved in the serotype switch event (Appendix 2). Phylogenetic analysis using publicly available genome sequences revealed single contig ST271 genomes that shared highest relatedness with 3/ST271. Strain A026 (19F/ST271) recovered in China during 2006–2008 was the most highly related putative genetic recipient (Figure, panel A). Two 19F/271 invasive ABCs isolates recovered from infants (6299-05 in Tennessee in 2004 and 2012214924 in California in 2012) had the closest matching PBP type to serotype-switch 3/ST271 isolates, sharing 2 of 3 PBP sequences (PBP2B-16 and PBP2x-47) (Appendix 1). Both ABCs 19F/ST271 isolate genomes shared more relatedness with 3/ST271 than publicly available 19F/271 genomes (Figure, panel A). By using BLAST (https://blast.ncbi.nlm.nih.gov/Blast.cgi) and whole-genome shotgun database (Appendix 2), we identified the likely *cps3* donors (3/ST700 strains B20605 and 73D368810). B20605 and 73D368810 shared sequence identity to the 9522 bp region (*dexB* to *pgm*) encompassing the ≈5,000-bp *cps3* operon of the 3/ST271 isolates. In contrast to serotype 3 strains in the United States that are typically basally susceptible to antimicrobial drugs (*4*), the likely serotype 3/ST700 donor strain was predicted to have intermediate penicillin resistance attributable to the mosaic *pbp1a* gene (PBP1A-17), which was co-transferred with the donor *cps3* locus to the 19F/ST271 recipient (Appendix 1). Serotype 3/ST700 isolates are documented in several countries in Africa but not in the United States (https://pubmlst.org/spneumoniae).

**Figure:**
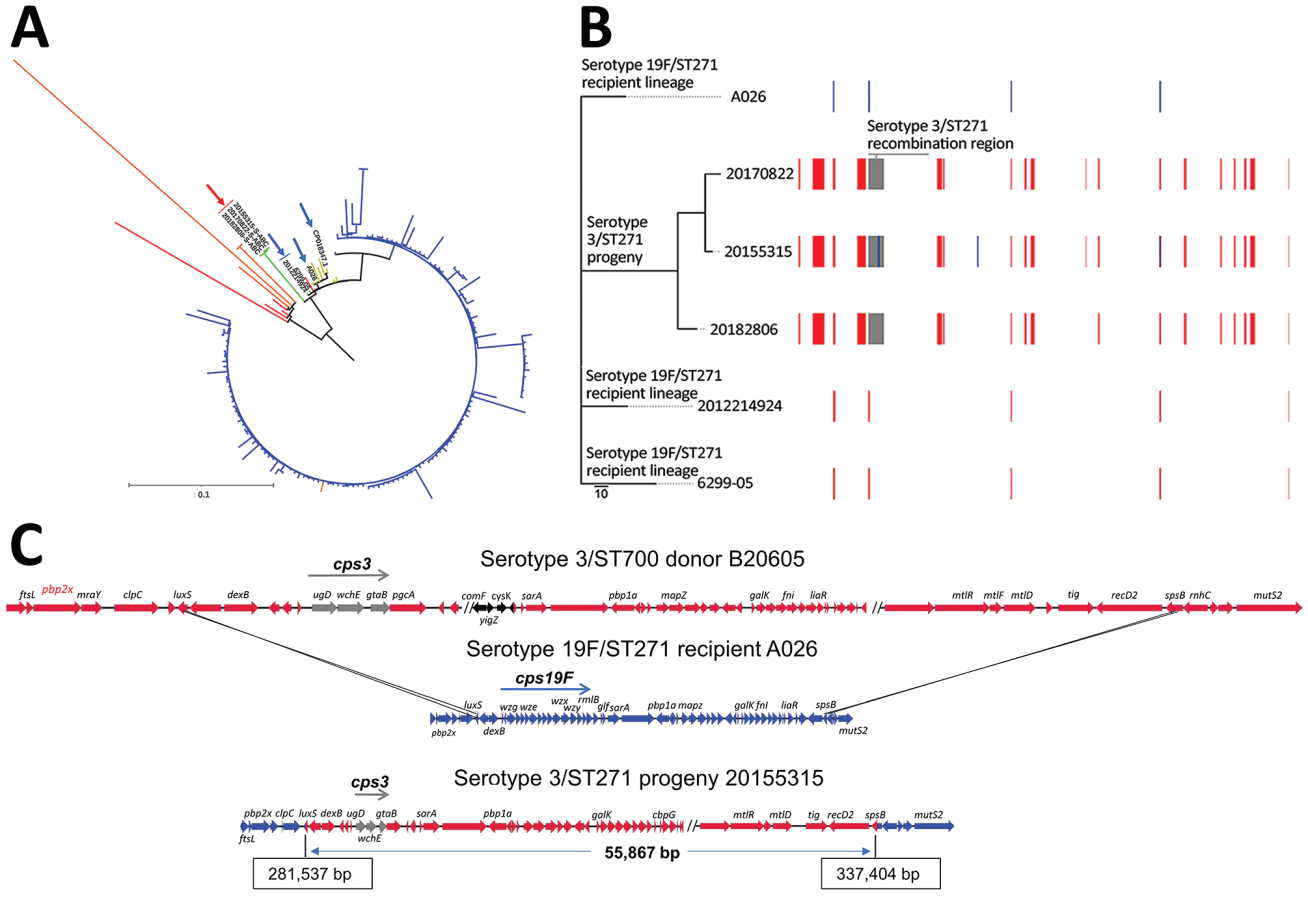
*Streptococcus pneumoniae* serotype 3/ST271 lineage resulting from a recombination event between a 19F/ST271 recipient and 3/ST700 donor. A) Phylogenetic tree showing progeny serotype 3/ST271 isolates 20155315-S-ABC, 20170822-S-ABC, and 20182806-S-ABC (red arrow) most closely related to the putative recipient 19F/ST271 isolates 6299-05 and 2012214924 (blue arrows). The most closely related ST271 single contig reference is A026 (also indicated with blue arrow). Branch colors: yellow, additional 19F/ST271 Active Bacterial Core surveillance (ABCs) isolates and single contig references; red, single-locus variant single contig references or ABCs isolates; orange, double-locus variant single contig reference or ABCs isolates; blue, ST320 ABCs isolates. Zero-, single-, and double-locus variant, single contig references were identified by using the PubMLST database (https://pubmlst.org). Scale bar corresponds to 1,062 single nucleotide polymorphisms. B) Phylogenetic alignment of the 3 recombinant serotype 3/ST271 isolates and closest known genomic matches of the ST271 recipient lineage and a schematic of recombinant genome fragments, represented by rectangular blocks, that were predicted by Gubbins (*10*). Block locations and sizes are relative to the aligned genomes; red blocks represent sites in common between >2 isolates (1 site, <150 bp in length, was not counted among the 17 total shown), blue blocks sites unique to a given isolate, and gray blocks the serotype-switch fragment that replaced the corresponding *cps19F* region within the recipient 19F/ST271 strain. The *cps3* locus was not identified using Gubbins because of its complete divergence from *cps19F* and instead was identified using ProgressiveMauve (*12*) within the encompassed 55.9 kb fragment (panel C). Gray block contains *cps3*, *pbp1a* (PBP1A-17), trigger factor, and choline binding protein G genes. C) Schematic illustrating ancestral recombination event between the 3/ST700 donor (B20605) and 19F/ST271 recipient (A026) to yield the 3/ST271 progeny (20155315-S-ABC). Deduced crossover points, including coordinates in the progeny, are shown. *luxS* and *spsB* genes are shown as blue/red hybrids. The minimum genes of the *cps3* operon required for polysaccharide capsuale biosynthesire shown in gray (*ugd, wchE, gtaB*). Genes with arrows in black differ between B20605 and 73D36881 and are absent in the 3/ST271 isolates. ST, sequence type.

To identify recombination sites, we mapped sequencing reads from the 3 3/ST271 isolates, 6299-05 (19F/ST271), and 2012214924 (19F/ST271) to A026 (19F/ST271) and then aligned the 6 genomes as described (*8*,*9*) (Appendix 2). The aligned 19F/ST271 and 3/ST271 genome sequences were input into Gubbins (*10*) (Appendix 2), which identified 17 recombinational fragments within 3/ST271 (median size 6,629 bp [range 363–59,159 bp]) (Figure, panel B). BEDTools (*9*) was applied to extract DNA sequences from recombinational fragments, and Prokka (*11*) was used to annotate sequences. Progressive Mauve (*12*) revealed the *cps3* operon in the 3 serotype 3/ST271 isolates on a 55.9-kb fragment that apparently originated from the serotype 3/ST700 donor (Figure, panel C). This region included the *cps3* operon, *pbp1a*, and 2 additional virulence factor genes (trigger factor [*tig*] and choline binding protein G [*cbpG*]). The corresponding region in 3/ST700 isolate B20605 contains genes that differ from serotype 3/ST700 isolate 73D36881 and are absent in the 3/ST271 isolates (Figure, panel C). This difference indicates that additional gene insertions occurred within this potential recombination hotspot in B20605 and 73D36881 and that this region subsequently might have been lost in the serotype 3/ST271 progeny.

When we compared the genes encoded in the serotype-switch region of the recipient with the corresponding region from the progeny and donor, we found they encoded near-identical virulence factors as identified by using the Virulence Factors Database (http://www.mgc.ac.cn/VFs/main.htm). The *tig* and *cbpG* genes have roles in pathogenesis and are cell wall surface–localized, highly conserved among *S. pneumoniae*, and immunogenic (*13*–*15*). Capsular polysaccharides provide the basis of approved *S. pneumoniae* vaccines. The limited efficacy of the serotype 3 component of PCV13 is probably attributable to high-level expression (thickness) of the serotype 3 capsule and its shedding by the bacteria (*6*,*7*). At the nucleotide level, *tig_20155315-S-ABC_* and *tig_A026_* share 99.1% identity (11 SNPs apart); *cbpG_20155315-S-ABC_* and *cbpG_A026_* share 51.0% identity, and *cps3* and *cps19F* operons are highly divergent (*5*). Moreover, the *pbp1a* gene within the 55.9-kb genomic fragment is identical between the 3 3/ST271 isolates and donor 3/ST700 strains and exhibits only 81.7% identity with the putative recipient 19F/ST271 strains. Recombination introduced a distinct virulence factor (replacement of the serotype 19F capsule with a structurally and serologically unique serotype 3 capsule) and introduced diversity in the surface protein virulence factors expressed in the serotype 19F/ST271 lineage.

To understand the effects of recombination, a comparison of fitness and virulence of progeny and parental strains in mouse models might be valuable. Our study highlights the existence of the 3/ST271 strain, because currently no vaccine is available and few antimicrobial drugs are predicted to optimally target this strain should it gain traction. Asymptomatic nasopharyngeal carriage serves as the major reservoir of highly common noninvasive infections and precedes invasive infections. Three nearly isogenic invasive isolates of this clone appearing over an expanse of 3 years in 2 different states is a concerning sign of a successful foothold within the carriage reservoir. All 3 cases were caused by bacteremic pneumonia in middle-aged patients. Of particular interest is the isolation of this strain 16 months apart from blood specimens from the same person (isolates 20155315 and 20170822), which is a rare occurrence and suggests a failure to eradicate the organism and long-term carriage or potentially the reacquisition of the strain from persons in the community. The recovery of still another invasive isolate of this same clone in 2018 further suggests a level of fitness required for long-term survival in the carriage reservoir and potential to cause IPD. Further, after the review process of this manuscript, ABCs has reported recovery of 2 additional closely related 3/ST271 case isolates during 2019 from Minnesota (B. Beall, unpub. data, 2020). This new strain complex has unique features potentially guiding its success, including high antimicrobial resistance, a capsule ineffectively targeted by PCV13, and undefined parameters inherent to the predominant invasive lineage of the post-PCV7 era. These observations emphasize the clear need for a pneumococcal vaccine that effectively targets serotype 3.

Appendix 1 Features of progeny, recipient, and donor strains for study of serotype-switch variant of multidrug-resistant Streptococcus pneumoniae sequence type 271.

Appendix 2 Additional methods for study of serotype-switch variant of multidrug-resistant *Streptococcus pneumoniae* sequence type 271.
