# Supplementary material for: Serotype-Switch Variant of Multidrug-Resistant Streptococcus pneumoniae Sequence Type 271
Source: Emerg Infect Dis. 2021 Jun;27(6):1689–92. doi: 10.3201/eid2706.203629 (PMC8153891; doi:10.3201/eid2706.203629)
Supplement: Appendix 2 — Additional methods for study of serotype-switch variant of multidrug-resistant Streptococcus pneumoniae sequence type 271. [file 20-3629-Techapp-s2.pdf]

Article DOI: https://doi.org/10.3201/eid2706.203629

Pneumococcal Serotype-Switch Variant of Multidrug-Resistant *Streptococcus pneumoniae* Sequence Type 271

Appendix 2

Methods

Identification of Donor and Recipient Strains

NCBI's Nucleotide BLAST (https://blast.ncbi.nlm.nih.gov/Blast.cgi) was used to find SPN isolates with highest identity to 3/ST271 cps3 operon (dexB to pgm), with whole genome-shotgun (wgs) database and *Streptococcus pneumoniae* organism (taxid: 1313) as search parameters.

kSNP3 version 3.0.0 (https://sourceforge.net/projects/ksnp/files) was applied to find the closest single contig reference to 3/ST271 isolates, with a Kchooser determined kmer of 23 and "–core" command line argument to calculate the phylogenetic tree based on SNPs found in all genomes.

Interactive Tree of Life (iTOL version 5; https://itol.embl.de) was used to visualize phylogenetic trees resulting from kSNP3 by re-rooting the tree to the branch containing single and double locus variant single contig references.

Figure, panel A was generated using kSNP3 and iTOL as described above. Figure, panel B was generated using Prokka annotations of indication regions and Easyfig (https://mjsull.github.io/Easyfig). Figure, panel C (https://wwwnc.cdc.gov/EID/article/27/6/20-3629-F1.htm) was generated using gubbins_drawer.py.

Read Mapping

Bowtie2 version 2.2.4 (https://github.com/BenLangmead/bowtie2) was applied to map whole genome sequencing reads from ABCs isolates 20155315, 20170822, 20182806, 6299–05 (ERR600161), and 2012214924 (ERR600302) to A026 with the "–very-sensitive-local" option.

Samtools suite version 0.1.18 (http://www.htslib.org) was applied to order the mapped reads with reference to A026 using the “view,” “sort” and “index” commands.

FreeBayes version 0.9.21 (https://github.com/ekg/freebayes) was used to align the indexed, sorted genome sequences of 20155315-S-ABC, 20170822-S-ABC, 20182806-S-ABC, ERR600161, ERR600302, and A026, with the “-j” (omit indels) and “- = ” options and specifying a base and mapping quality of 20 (“-q 20” and “-m 20”).

Vcflib suite version 2015.10.19 (https://github.com/vcflib/vcflib#vcflib) was applied to remove weakly called variants in the read mapping alignment file generated by Bowtie2, Samtools, and Freebayes with the vcffilter command, and the following “-f” and “-g” options: -f “QUAL >200 & DP >15 & TYPE = mnp | QUAL >200 & DP >15 & TYPE = snp” -g “GQ >30 & AO >10 & GT = 1”

Bedtools suite version 2.17.0 (https://github.com/arq5x/bedtools2) genomecov utility was applied to generate a file describing regions of low coverage (<10x) with the “-bga” option. The maskfasta utility was used to mask out all low coverage regions with N’s.

Gubbins Recombination Analysis

The input for Gubbins version 2.3.1 (https://github.com/sanger-pathogens/gubbins) was aligned sequences (20155315-S-ABC, 20170822-S-ABC, 20182806-S-ABC, ERR600161, ERR600302, and A026) where low coverage regions had been masked with N’s. Default settings were used with the “-i” option set for 20 iterations and with A026 specified as the outgroup. The gubbins_drawer.py command was used to generate a PDF of the putative recombinant regions using the output .tre and .embl files.

A .bed file was generated from the recombination sites’ coordinates using the Gubbins output .gff file.

Bedtools suite version 2.17.0 getfasta utility was used with this .bed file to generate an output file with all of the recombination site sequences extracted from the original Gubbins input file. NCBI’s Nucleotide BLAST was then used to identify SPN isolates with highest identity to these recombination sites, with nucleotide collection (nr/nt) database and *Streptococcus pneumoniae* organism (taxid: 1313) as search parameters. Recombinant sites <300 bp in length

(n = 3; 121 bp, 36 bp, and 110 bp) were omitted from the analysis. No regions with identity to serotype 3 were found.

Extracted regions were also annotated using Prokka version 1.14.3 (https://github.com/tseemann/prokka) with "–addgenes" and "–compliant" options; however, a cps3 operon was not identified.

Progressive Mauve Recombination Analysis and Prokka Annotation

Abacas version 1.0 (http://abacas.sourceforge.net) was used to sort and index whole genome assemblies from 20155315-S-ABC, 20170822-S-ABC, 20182806-S-ABC, ERR600161, and ERR600302 with reference to A026 with the "-m," "-N," and "-a" options.

Aligned genomes were annotated with Prokka version 1.14.3 using "–addgenes" and "–compliant" options with and without "–genus Streptococcus" and "–species pneumoniae" options.

Prokka annotations were input into progressiveMauve version 2.4.0 (http://darlinglab.org/mauve/download.html). Initially the cps3 operon and ultimately the ~55.9kb recombination site were identified by manually searching for regions of dissimilarity between the three 3/ST271 isolates 20155315-S-ABC, 20170822-S-ABC, 20182806-S-ABC and the two 19F/ST271 isolates ERR600161 and ERR600302 within the appended sequences ("-a" option in Abacas).

Pairwise alignments were conducted using EMBOSS Needle (https://www.ebi.ac.uk/Tools/psa/emboss_needle) or Clustal Omega (https://www.ebi.ac.uk/Tools/msa/clustalo).

GenBank Accession Numbers

Isolates were submitted to GenBank (accession nos. 73D36881, B20605 (NZ_FIXO01000000, NZ_FIXN01000000), A026 (CP006844.1).
